# Evaluation of Cardiac Function in a Patient With Hypertrophic Cardiomyopathy Before and During Pregnancy to Predict Its Outcome: A Case Report

**DOI:** 10.7759/cureus.81124

**Published:** 2025-03-24

**Authors:** Yuta Shimomoto, Ryuhei Nagai, Yusuke Ujihara, Nagamasa Maeda

**Affiliations:** 1 Obstetrics and Gynecology, Kochi Medical School, Kochi, JPN

**Keywords:** hypertrophic cardiomyopathy, maternal prognosis, neonatal prognosis, pregnancy and heart disease, prognostication methods

## Abstract

The risk of maternal and neonatal mortality in pregnant women with hypertrophic cardiomyopathy (HCM) is considered low, and prognostic methods have not yet been established. In this study, we evaluated several pregnancies in the same patient. In each case, we were able to assess the severity of the mother's cardiac disease and estimate the prognosis. By reviewing the severity of the disease over time and its actual course, we verified the kind of assessment that is important for predicting prognosis. We present the case of a patient who was diagnosed with HCM at 18 years of age and had her first spontaneous pregnancy at 25 years of age. The baby was born at 38 weeks and was healthy, but the mother's HCM worsened after the second trimester of pregnancy, and treatment in the intensive care unit was needed after delivery. After an implantable cardioverter-defibrillator (ICD) implantation, a second pregnancy was established. However, due to repeated arrhythmias requiring ICD activation, a termination of pregnancy was performed due to the risk of worsening maternal heart failure. After radical septal myectomy, a third pregnancy was achieved. In this case, the left ventricular outflow tract stenosis disappeared, the left ventricular outflow tract pressure gradient decreased, and the pregnancy progressed well with no maternal complications. The New York Heart Association (NYHA) and modified World Health Organization (WHO) scores before conceiving were not sufficient to predict pregnancy outcomes. Changes in the disease status during pregnancy were generally consistent with the Zwangerschap bij Aangeboren HARtAfwijkingen (ZAHARA) or pregnancy and congenital heart disease score, Cardiac Disease in Pregnancy Study II (CARPREG II) score, and modified WHO classification ratings, suggesting that they are suitable for assessing risk during pregnancy, and before and after delivery in patients with HCM.

## Introduction

Hypertrophic cardiomyopathy (HCM) is a group of diseases characterized by hypertrophy of the left or right ventricular myocardium and decreased left ventricular diastolic function due to cardiac hypertrophy [1]. HCM increases the risk of heart failure, thrombosis (embolic stroke), atrial and ventricular arrhythmias, and sudden death but is often asymptomatic [2]. Although HCM is considered a rare disease, according to a recent survey, its prevalence in Japan was reported to be approximately 1.8% [3]. Therefore, HCM may be more common than previously thought, and its association with pregnancy should always be considered in young patients. During pregnancy, the treatment of HCM focuses on managing symptoms while ensuring the safety of both the mother and the baby. Beta-blockers are commonly used as they are generally considered safe during pregnancy. Diuretics and antiarrhythmic drugs may be used cautiously if needed. Invasive procedures like septal myectomy or alcohol septal ablation are usually avoided during pregnancy.

Pregnancy causes several changes in maternal hemodynamics, including increased cardiac output, decreased systemic vascular resistance, activation of the renin-angiotensin-aldosterone system, and increased heart rate, which could theoretically increase the maternal cardiac load, the risk of heart failure, and arrhythmia. Studies have shown that many pregnant women with HCM have uneventful pregnancies with no maternal or infant mortality [2-4]. However, the risk of cardiovascular events during pregnancy is increased in patients with HCM, resulting in moderate to severe systolic or diastolic dysfunction, severe left outflow tract obstruction, or pulmonary hypertension [5,6]. In these cases, it is ideal to predict prognosis before pregnancy. Moreover, since cardiac function is expected to change during pregnancy, it is desirable to have a method to evaluate prognosis over time. However, evidence-based prognostic indicators have not yet been identified.

In this case study, pregnancies complicated by HCM with different pregnancy outcomes were observed in the same patient. This was a case of a female patient with heart disease who required multidisciplinary treatment due to the exacerbation of heart failure during pregnancy, despite being in a category that allowed her to continue her pregnancy. This was based on the modified World Health Organization (WHO) and New York Heart Association (NYHA) classifications, which are commonly used as indicators for granting permission for pregnancy in such patients. What is particularly noteworthy is that the evaluation before conception with these classifications allowed for the continuation of both the pregnancies. However, in one pregnancy, the patient's condition worsened and intensive treatment was required, whereas in the other, it progressed well. The course of the pregnancies was reviewed and the laboratory data obtained from the medical records were retrospectively reevaluated to determine whether it was possible to predict the disease status of HCM and the prognosis of the mother and the fetus.

## Case presentation

A 25-year-old female patient presented to our institution with a family history of her mother with HCM and an uncle (mother’s brother) with a history of cardiopulmonary arrest. The patient had been diagnosed since elementary school with a heart murmur and an abnormal electrocardiogram. At 18 years of age, she was also diagnosed with an abnormal electrocardiogram at an entrance examination for a vocational school and was referred to another medical institution. The patient was then referred to our cardiology department. Close examination with an echocardiogram revealed an asymmetric enlargement of the ventricular septum and left ventricular hypertrophy, along with the family history leading to a diagnosis of HCM. The mother was also diagnosed with HCM and information regarding genetic testing was provided. However, consent was not obtained, and no testing was performed. The patient was managed conservatively, and metoprolol tartrate was used as an abortive treatment for dyspnea and other symptoms. Regular follow-up visits were made to a local internal medicine clinic but she had not received permission to become pregnant. However, spontaneous pregnancy was established. She was referred to the perinatal center at eight weeks of pregnancy. 

Progression of the first pregnancy

**Figure 1:**
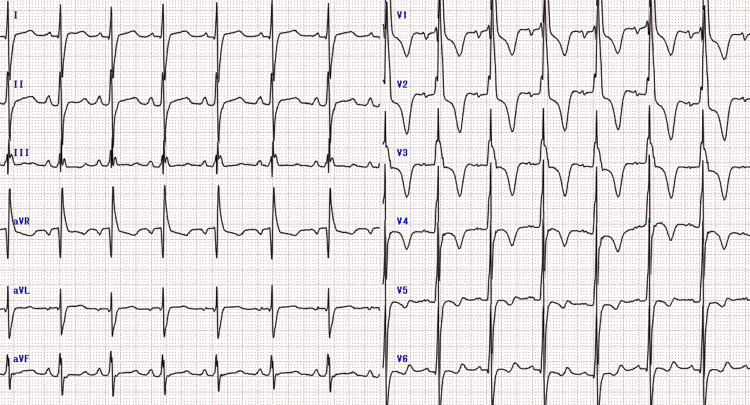
The first ECG carried out during early pregnancy There is a high potential in the left ventricle, indicating left ventricular overload. There is no abnormal Q wave, QRS prolongation, or arrhythmia.

**Figure 2:**
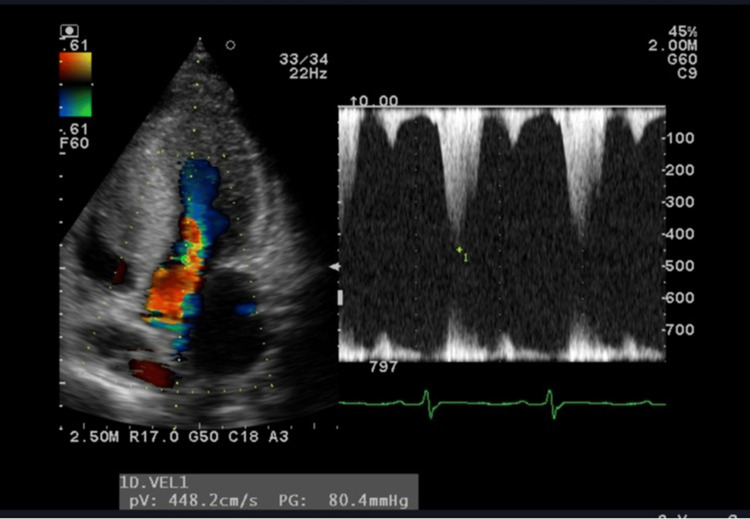
Echocardiography early in the first pregnancy The left ventricular outflow tract pressure gradient was as high as 80 mmHg.

The patient's HCM was evaluated using the NYHA classification. The degree of left ventricular outflow tract stenosis was evaluated by echocardiography, and valvular status and arrhythmia via an electrocardiogram. In early pregnancy, the NYHA classification was grade II and the echocardiography showed severe left ventricular outflow tract stenosis with a left ventricular outflow tract pressure gradient of 80 mmHg (a left ventricular outflow tract pressure gradient of 50 mmHg or greater was considered significant) (Figures 1, 2) and mild mitral regurgitation as valvular disease.

**Figure 3:**
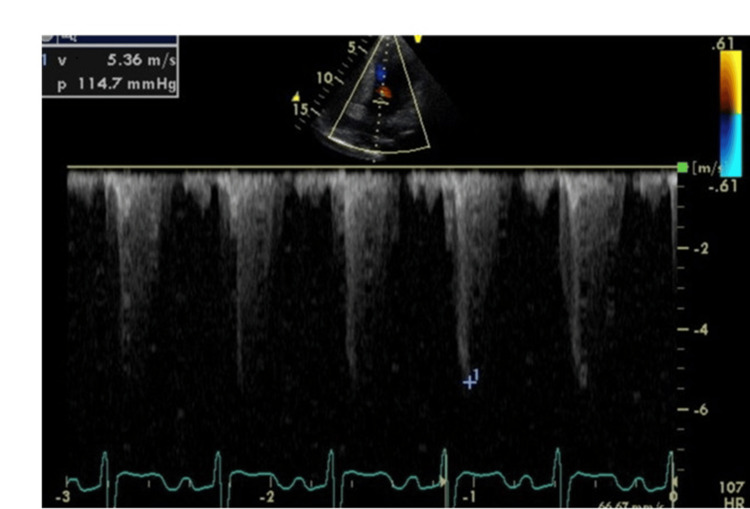
Echocardiography at 24 weeks of the first pregnancy The left ventricular outflow tract pressure gradient worsened to 114 mmHg.

Electrocardiography revealed no arrhythmias. Echocardiography at 24 weeks of gestation revealed an increased left ventricular outflow tract pressure gradient of 114 mmHg, worsening left ventricular outflow tract stenosis (Figure 3), and moderate to worsening mitral regurgitation.

Holter electrocardiography at 28 weeks of gestation showed three consecutive instances of non-sustained ventricular tachycardia, which had not been previously reported. In HCM, three or more consecutive episodes of non-sustained ventricular tachycardia constitute moderate arrhythmia. In the second trimester of pregnancy, the NYHA of the patient worsened from grade II to III. Complete right bundle branch block on electrocardiography and cardiac enlargement with a cardiothoracic area ratio (CTAR) of 62% on chest radiography (CTAR value had not changed since before pregnancy, but there was cardiac enlargement), which represent worsening heart failure, were both observed in the patient. Considering the cardiac enlargement, the high risk of arrhythmia during vaginal delivery, and the possibility that maternal circulatory dynamics could not be maintained when arrhythmia occurred, an elective cesarean section at 38 weeks 0 days of gestation was decided as the delivery method. Spinal subarachnoid anesthesia was not used to avoid a sudden intraoperative decrease in preload. Instead, epidural anesthesia was administered. Intraoperatively, the delivery of the fetus and placenta was smooth; however, the patient complained of dyspnea after intravenous and local uterine administration of oxytocin. Suspecting supine hypotensive symptoms, the position of the patient was changed to semi-sitting, and oxygen administration was initiated. However, the drop in oxygen saturation (SpO2) did not improve. As the mean blood pressure decreased, a bolus of 0.05 mg of phenylephrine was administered, but this had no impact on the SpO2. An echocardiographic examination was performed to investigate the cause of low SpO2 and dyspnea. It revealed circumferential left ventricular systolic dysfunction and tricuspid regurgitation (mild-to-moderate). Postoperative chest radiography showed a CTAR of 62%, cardiac enlargement, and pulmonary congestion, which were judged to indicate dyspnea and low SpO2 due to the acute exacerbation of chronic heart failure.

**Figure 4:**
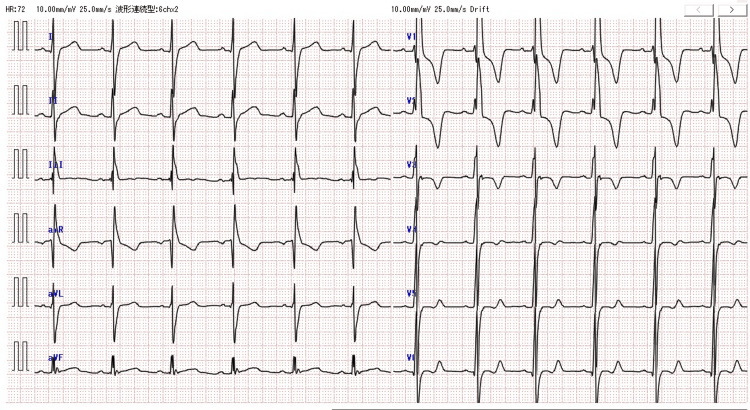
ECG after the first delivery Compared to early pregnancy, although no arrhythmia was observed, an increase in left ventricular load, an increase in T waves, and an extension of QRS was observed.

Postoperatively, the patient was admitted to the intensive care unit for the management of heart failure. Oxygen administration was initiated at 30 L/min with a high nasal flow rate, and SpO2 was maintained at 99%. A bolus dose of 10 mg furosemide was administered to treat pulmonary edema. On the first postoperative day, chest radiography revealed a CTAR of 67%, worsening cardiac enlargement, and pulmonary congestion that had not improved. However, SpO2 was maintained at 99% even when the oxygen dosage was reduced to 5 L/min of mask oxygen. At this point, the patient was still poorly oxygenated with an arterial oxygen partial pressure (PaO2) to the fraction of inspired oxygen (FiO2) ratio or P/F ratio of 260; therefore, she continued to be managed in the intensive care unit. On the second postoperative day, oxygen was administered at 3 L/min of mask oxygen. SpO2 was stable at 99%, chest radiography showed a slight improvement with a CTAR of 62%, the pulmonary congestion improved, and the P/F ratio was 318, all of which indicated improved oxygenation. Oxygen administration was gradually tapered off. On the fifth postoperative day, SpO2 was maintained at 99% and oxygen administration was terminated. The NYHA of the patient improved from IV to III. On the seventh postoperative day, propranolol hydrochloride was administered to treat HCM. At the one-month checkup, her NYHA score had improved to II (Figure 4). 　

Progression of the second pregnancy

**Figure 5:**
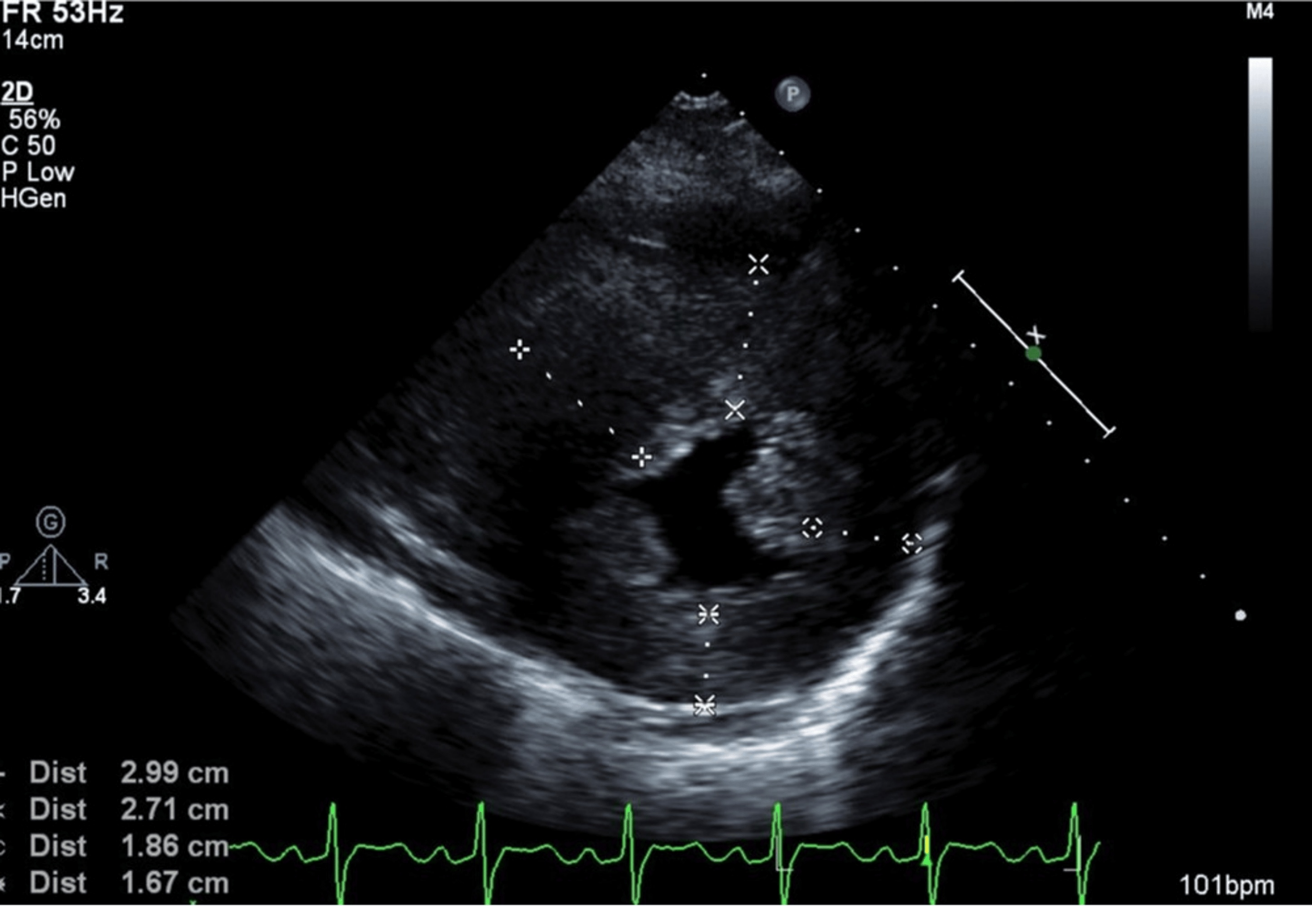
Echocardiography during the course of the first pregnancy The thickness of the left ventricular wall had reached almost 30 mm.

During the course of the first pregnancy, non-sustained ventricular tachycardia on Holter ECG and left ventricular wall thickness greater than 30 mm on echocardiography (Figure 5) were observed, both of which were risk factors for lethal arrhythmia in HCM.

**Figure 6:**
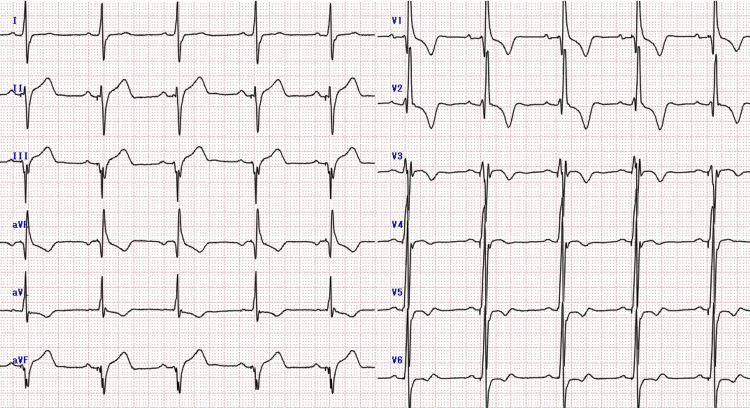
ECG during the first trimester of the second pregnancy The left ventricular load increased, the T wave increased, and the QRS remained unchanged. The ICD was activated by ventricular fibrillation occurring immediately with light movement.

In addition, the risk of sudden death due to HCM arrhythmia as denoted by the HCM Risk-SCD score, showed a high risk (8.87%). Therefore, an implantable cardioverter-defibrillator (ICD) implantation was performed nine months after delivery. However, even after implantation, the patient developed ventricular fibrillation with light exertion (Figure 6), and the ICD was activated.

**Figure 7:**
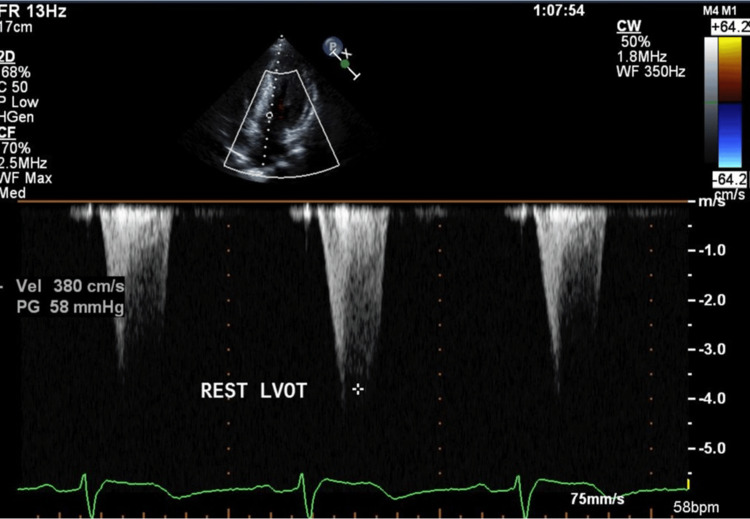
Echocardiography during the second pregnancy The left ventricular outflow tract pressure gradient was as high as 58 mmHg.

Although her NYHA score was grade II at the beginning of the pregnancy, echocardiography revealed a high left ventricular outflow tract pressure gradient of 58 mmHg (Figure 7), left ventricular outflow tract stenosis, and mitral regurgitation (mild). 

The patient was very keen on continuing the pregnancy. However, her obstetrician and cardiologist were concerned that her condition was not being sufficiently controlled with anti-arrhythmic drugs, and that, as with the first child, if chronic heart failure were to worsen acutely during the pregnancy, the mother's life could be at risk. After discussing the risks of continuing the pregnancy completely with the patient and her family, it was terminated.

Progression of the third pregnancy

**Figure 8:**
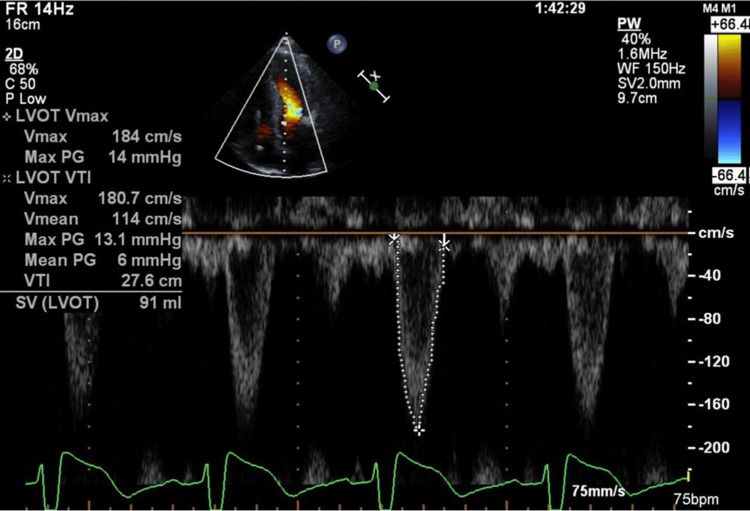
Echocardiography before the third pregnancy The left ventricular outflow tract pressure gradient had improved to 6-13 mmHg.

After the second pregnancy ended, the arrhythmia and heart failure symptoms could not be controlled using drug therapy. The patient requested a surgical procedure to improve her heart failure symptoms to allow her to conceive. Postoperative echocardiography showed improvement in left ventricular outflow tract stenosis (left ventricular outflow tract pressure range 6-13 mmHg) (Figure 8).

Thereafter, the patient was treated with bisoprolol fumarate tablets alone. Based on her postoperative course, the cardiologist allowed her to conceive, and she conceived spontaneously.

**Figure 9:**
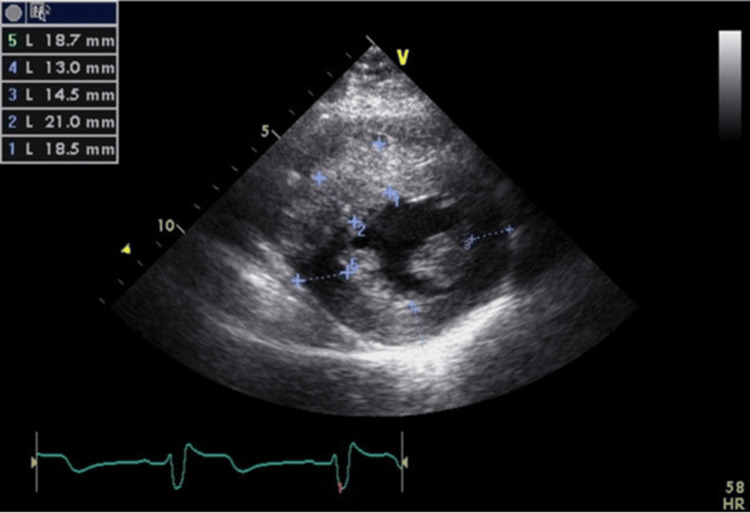
Echocardiography during the third pregnancy The maximum thickness of the left ventricular wall was 21 mm.

**Figure 10:**
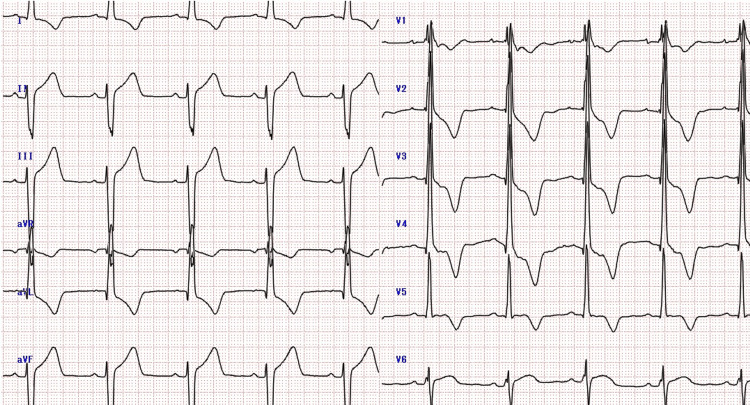
ECG during the first trimester of the third pregnancy Although an increase in T-wave was observed, there were no findings of left ventricular load, no arrhythmia, and the ICD did not get activated.

At the beginning of pregnancy, her NYHA class was II, echocardiography showed no left ventricular outflow tract stenosis, and ICD monitoring showed no arrhythmias (Figures 9, 10).

Echocardiography was performed every month throughout the pregnancy, and no left ventricular outflow tract stenosis was observed. The patient underwent a repeat cesarean section, and general anesthesia was chosen. This was because, despite opting for epidural anesthesia (rather than spinal anesthesia) to avoid sudden circulatory changes during the first cesarean section, the patient’s chronic heart failure and pulmonary edema had acutely worsened due to inadequate management. An elective cesarean section was performed under general anesthesia at 37 weeks of gestation. No significant changes in vital signs were observed during or after the operation. Immediately after surgery, the patient was monitored in the intensive care unit. However, her general condition remained stable, and she was transferred to the general ward on the first postoperative day. Her subsequent progression was good and she was discharged on the eighth postoperative day. The patient did not have any apparent cardiovascular events during the entire pregnancy period up to one month postpartum and progressed well.

**Figure 11:**
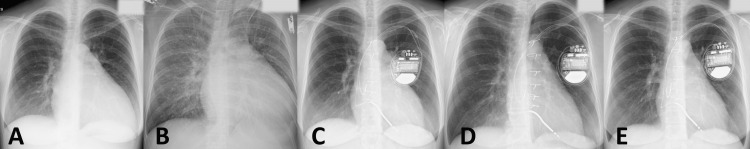
Chest X-ray (time series display) A: Before the first pregnancy: Cardiothoracic area ratio (CTAR) of 62%, and cardiac enlargement is observed. B: After the first cesarean delivery: Poor lung field permeability and cardiac enlargement are observed (CTAR 67%). C: Before the second pregnancy: After implantable cardioverter-defibrillator (ICD) implantation, cardiac enlargement is not very noticeable (CTAR 50%). D: Before the third pregnancy. E: After the third delivery: It is almost the same as before the pregnancy.

The figure below shows a time series display of the cardiac enlargement occurring across the three pregnancies (Figure 11). 

Risk assessment

Since the same patient experienced different pregnancy outcomes, the risk of cardiovascular events in HCM-complicated pregnancies was evaluated retrospectively using three endpoints: the Zwangerschap bij Aangeboren HARtAfwijkingen (ZAHARA) risk score, the Cardiac Disease in Pregnancy Study II (CARPREG II) score and the modified World Health Organization (WHO) classification.

The ZAHARA risk score predicts maternal cardiovascular events using eight risk factors based on a retrospective cohort study of 714 pregnancies complicated by congenital heart disease conducted in 2010 [7]. The CARPREG II score was used to predict maternal cardiovascular events using 10 risk factors based on a cohort study of 1,938 pregnant women with heart disease in 2018 [8]. The modified WHO classification is a risk score proposed by the ESC (European Society of Cardiology) guidelines, which includes an assessment of each disease in addition to its clinical status [9].

In the present case, the risk assessment was performed before the first pregnancy, late in the first pregnancy, before second pregnancy, before the third pregnancy, and at the end of the third pregnancy (Table 1).

**Table 1:** Changes in each score over time Data presented in bold indicates high risk in each risk assessment (ZAHARA score: 3.5 or higher, CARPREG II score: 5 or higher, mWHO: Ⅳ or higher, NYHA: 4). ZAHARA: Zwangerschap bij Aangeboren HARtAfwijkingen; CARPREG: Cardiac Disease in Pregnancy Study; mWHO: modified World Health Organization; NYHA: New York Heart Association

	Pre-pregnancy	Late during the first pregnancy	Before second pregnancy	Before third pregnancy	Late during the third pregnancy
ZAHARA	4	7	6.25	3	3
CARPREG Ⅱ	5	8	5	3	3
mWHO	Ⅱ-Ⅲ	Ⅳ	Ⅱ-Ⅲ	Ⅱ-Ⅲ	Ⅱ-Ⅲ
NYHA	2	4	2	2	2

Risk Assessment During the First Pregnancy

Before the first pregnancy, the patient had a ZAHARA score of 4.0 (pre-pregnancy medical therapy: 1.5, left ventricular outflow tract stenosis: 2.5), a CARPREG II score of 2 (left ventricular outflow tract stenosis: 2), and a modified WHO classification of II-III (HCM). Maternal cardiovascular event rates were 70% for the ZAHARA score, 8% for the CARPREG II score, and 10-19% for the modified WHO classification. In late pregnancy, her NYHA worsened from grade II to III, Holter electrocardiography showed non-sustained ventricular tachycardia, and echocardiography showed mitral regurgitation in the mild-to-moderate range. The ZAHARA score in the second trimester was 7.0 (history of arrhythmia: 1.5, pre-pregnancy medications: 1.5, NYHA≥III: 0.75, left ventricular outflow tract stenosis: 2.5, and atrioventricular valve regurgitation: 0.75) and the CARPREG II score was 8 (pre-pregnancy cardiovascular events: 3, NYHA≥III: 3, left ventricular outflow tract stenosis: 2), and a modified WHO classification of IV (NYHA grade III). The incidence of maternal cardiovascular events in late pregnancy was 70% for the ZAHARA score, 40-45% for the CARPREG II score, and 40-100% for the modified WHO classification, with the highest risk for any score.

Risk Assessment During the Second Pregnancy

The ZAHARA score was 6.25 (history of arrhythmia: 1.5, pre-pregnancy medical therapy: 1.5, left ventricular outflow tract stenosis: 2.5, and atrioventricular valve regurgitation: 0.75); the CARPREG II score was 5 (cardiovascular events before pregnancy: 3, left ventricular outflow tract stenosis: 2), and the modified WHO classification was II-III (HCM). The maternal incidence of cardiovascular events was high risk: 70% according to the ZAHARA score, 40-45% according to the CARPREG II score, and 10-19% according to the modified WHO classification.

Risk Assessment During the Third Pregnancy

The risk assessment before pregnancy according to the ZAHARA score was 3.0 (history of arrhythmia: 1.5, medical therapy before pregnancy: 1.5), according to the CARPREG II score was also 3 (cardiovascular events before pregnancy: 3), and II-III (HCM) according to the modified WHO classification. Maternal cardiovascular event rates were 43.1% for the ZAHARA score, 15% for the CARPREG II score, and 10-19% for the modified WHO classification.

## Discussion

HCM is a group of disorders characterized by left or right ventricular myocardial hypertrophy and decreased left ventricular diastolic function due to cardiac hypertrophy, which may be associated with heart failure and arrhythmias [10]. The management of HCM complicated by pregnancy is an issue before and after conception, but there is a paucity of literature describing the evaluation of disease status, as well as prognosis before and during pregnancy. In this study, we observed different outcomes in the same patient with HCM. In this case, the patient experienced three pregnancies, each with a different course. We therefore retrospectively evaluated the risk of cardiovascular events during pregnancy using three risk scores.

Although NYHA is usually used to assess the risk of pregnancy in patients with heart failure and those with HCM, left ventricular outflow tract stenosis, supraventricular or ventricular arrhythmias, and valvular disease associated with HCM should also be assessed. We hypothesized that the ZAHARA score, CARPREG II score, and modified WHO classification could be used to evaluate and predict the outcome of pregnancies complicated by HCM, as the ZAHARA score includes risk factors such as a history of arrhythmia, medical therapy before pregnancy, and mitral regurgitation, which are suitable for the evaluation of HCM-complicated pregnancies (Tables 2, 3) [7].

**Table 2:** ZAHARA risk score *Pressure gradient >50 mmHg or aortic valve mouth area <1.0 cm^2^. The ZAHARA risk score predicts maternal cardiovascular events using eight risk factors. ZAHARA: Zwangerschap bij Aangeboren HARtAfwijkingen; NYHA: New York Heart Association; AV: Atrioventricular

Risk factors for maternal cardiovascular events	Score
History of arrhythmia	1.50
Medical therapy before pregnancy	1.50
NYHA≥Ⅲ	0.75
Left ventricular outflow tract stenosis^*^	2.50
Atrioventricular valve regurgitation of body circulation	0.75
Pulmonary circulation AV valve regurgitation	0.75
Mechanical valve	4.25
Cyanotic disease	1.00

**Table 3:** ZAHARA risk scores and corresponding maternal cardiovascular event rates ZAHARA: Zwangerschap bij Aangeboren HARtAfwijkingen

Risk score value	Maternal cardiovascular event rate
0-0.50	2.9%
0.51-1.5	7.5%
1.51-2.5	17.5%
2.51-3.5	43.1%
>3.5	70.0%

The CARPREG II score includes left ventricular outflow tract stenosis and arrhythmias as risk factors and, like the ZAHARA score, is suitable for the evaluation of pregnancies complicated by HCM (Tables 4, 5) [8]. The diagnosis of HCM is 2-3, which is moderate risk, and NYHA III or higher is 4, which is ultra-high risk.

**Table 4:** CARPREG II Score The CARPREG II score was used to predict maternal cardiovascular events using 10 risk factors. CARPREG: Cardiac Disease in Pregnancy Study; NYHA: New York Heart Association; LVEF: Left ventricular ejection fraction

Risk predictors	Score
Cardiovascular event or arrhythmia before pregnancy	3
NYHA≥Ⅲ, cyanosis	3
Post-mechanical valve replacement	3
LVEF< 40%	2
Severe left ventricular inflow valve or outflow tract stenosis	2
Complicated pulmonary hypertension	2
Complicated coronary artery disease	2
Severe aortopathy	2
Previously untreated lesions	1
Patients with delayed pregnancy evaluation	1

**Table 5:** CARPREG II risk scores and the corresponding maternal cardiovascular event rates CARPREG: Cardiac Disease in Pregnancy Study

Risk score value	Maternal cardiovascular event rate
0-1	5%
2	8%
3	15%
4	20-25%
≥5	40-45%

In the case of NYHA III-IV, as in the present case, the modified WHO classification can be used to evaluate patients at high risk (Table 6) [9].

**Table 6:** Modified WHO Classification The modified WHO classification is a risk score proposed by the ESC (European Society of Cardiology) guidelines, which includes an assessment of each disease in addition to its clinical status. WHO: World Health Organization; PS: pulmonary stenosis; PDA: patent ductus arteriosus; MVP: mitral valve prolapse; APC: supraventricular extrasystole; VPC: ventricular extrasystole; CHD: congenital heart defect; ASD: atrial septal defect; VSD: ventricular septal defect; TOF: tetralogy of Fallot; EF: ejection fraction; HCM: hypertrophic cardiomyopathy; AVSD: atrioventricular septal defect; MVP: mitral valve prolapse; MS: mitral stenosis; CoA: coarctation of the aorta; AS: artic stenosis; NYHA: New York Heart Association; PAH: pulmonary arterial hypertension.

modified WHO classification	Cardiovascular complication rate	Maternal mortality risk	Maternal risk factors
I	2.5 to 5%	-	Minor PS, PDA, MVP, isolated APC, or VPC; Simple CHD after repair (ASD, VSD, PDA, pulmonary venous return abnormality)
II	5.7 to 10.5%	Mild	Unrepaired ASD, VSD, post-repair TOF; Most arrhythmias; Turner syndrome without aortic enlargement
II-III	10 to 19%	Moderate	Mild left ventricular dysfunction (EF>45%); HCM, Post-repair CoA; AVSD; Marfan syndrome without aortic enlargement; Aortic bicuspid valve with aortic diameter <45 mm; Autologous or bioprosthetic valvular disease other than WHO classification I/IV
III	19 to 27%	Severe	Moderate left ventricular dysfunction (EF 30-45%); Right ventricular support of the systemic circulation; Unrepaired cyanotic CHD; moderate MS; Mechanical valve; Good Fontan circulation, Other complex CHD; Ventricular tachycardia; Marfan syndrome with aortic diameter 40-45 mm; Aortic bicuspid valve with aortic diameter 45-50 mm; Pre-existing peripartum cardiomyopathy without left ventricular dysfunction
IV	40 to 100%	Very high. Contraindication to pregnancy	Severely depressed ventricular function (LVEF <30%, NYHA III-IV); Moderate to severely dysfunctional right ventricle; PAH; Severe MS; Symptomatic severe AS; Unrepaired severe CoA; Fontan circulation with complications; Marfan syndrome with aortic diameter >45 mm; Aortic bicuspid valve with aortic diameter >50 mm; Peripartum cardiomyopathy with residual left ventricular dysfunction

First pregnancy

All the three risk assessments (ZAHARA score, CARPREG II score, and modified WHO classification) were elevated early in the first pregnancy, suggesting a high rate of cardiovascular events during the pregnancy. In particular, the echocardiographic findings of left ventricular outflow tract stenosis (increased left ventricular outflow tract pressure gradient of 114 mmHg), valvular disease, and triplicate non-sustained ventricular tachycardia on Holter ECG increased the risk for each assessment. In practice, however, the only way to assess disease status at the time was by NYHA classification, echocardiography, and the presence of arrhythmias, making it difficult to objectively predict maternal risk during the pregnancy. Moreover, it was insufficient to consider whether the pregnancy should be continued. Although it is debatable whether the patient should have chosen to terminate the pregnancy if objective indicators were available, it is possible that both the patient and the healthcare provider could have had the opportunity to recognize the risks of continuing the pregnancy at this point and consider a course of action. In addition, all three risk assessments worsened to the highest risk rating in the second trimester of pregnancy. However, it was difficult to predict the worsening of the medical condition using existing assessment methods. It is also impossible to ignore the fact that the condition of the mother clearly worsened before and after delivery, requiring intensive care and became life-threatening. If the three risk assessments had been used, the risk could have been assessed over time, and therapeutic intervention could have been considered at the point of worsening trend or the highest risk. More importantly, although the patient had been diagnosed with HCM prior to conception, neither the providers nor the patients were fully informed about the maternal risks of pregnancy. In fact, the first pregnancy was a life-threatening situation for both the mother and child. Fortunately, the situation was resolved without incident. For women with HCM who are able to conceive, it is extremely important to use the three risk assessments to predetermine the risks of pregnancy and predict the prognosis so that both the patient and her healthcare provider can take action to avoid maternal risk. 

Second pregnancy

In the first trimester of her second pregnancy, the NYHA of the patient was grade II, and echocardiography showed a high left ventricular outflow tract pressure gradient of 58 mmHg. However, there was no deterioration compared to the first trimester, and there was no significant change in left ventricular outflow tract stenosis or mitral regurgitation (mild). Since there was no change in the subjective symptoms, the patient's desire to continue her pregnancy was very strong. As with the first pregnancy, there was still a lack of medical information to support the decision to continue the pregnancy. One difference was that the information from the previous pregnancy was shared by the patient's family and healthcare providers, and the assumed high maternal risk of the new pregnancy may have greatly influenced the decision-making process among the patient's family, obstetrician, and cardiologist. However, both the ZAHARA and CARPREG II scores were higher than the early pregnancy risk assessment of the first pregnancy (Table 1), indicating that the risk was as high as or even higher than the first pregnancy. The existence of an objective risk score is important. We had experienced a difficult course with the first pregnancy and elected to terminate the second pregnancy. If there had been a risk score available, it would have allowed us to determine whether to continue the pregnancy without relying on the experience of the first pregnancy.

Third pregnancy

The third pregnancy was established after septal myectomy to improve the HCM-induced heart failure symptoms and reduce the risk of cardiovascular events during pregnancy. However, the risk of cardiovascular events in the left ventricular flow tract of the mother was still potentially high. During the course of the third pregnancy, there was no worsening of NYHA, echocardiographic evidence of left ventricular outflow tract stenosis, or mitral regurgitation.

The symptoms of heart failure appeared after continuous intravenous oxytocin infusion and local uterine administration after delivery of the placenta during the cesarean section of the first child. Oxytocin decreases blood pressure due to decreased peripheral vascular resistance and increases cardiac output from the increased preload due to uterine contractions, which may have resulted in the induction of cardiac failure [11]. In the third pregnancy, continuous oxytocin infusion and local uterine administration were administered during the cesarean section, but there was no worsening of cardiac failure because the myocardial ablation resulted in antegrade heart failure. We believe that the use of oxytocin should be considered in pregnancies complicated by HCM when there is a high risk of exacerbation of heart failure associated with impaired myocardial dilatation.

Anesthesia for cesarean section in HCM is a matter of debate as a decreased preload decreases cardiac output by increasing the left ventricular outflow tract pressure gradient, whereas an excessive preload decreases cardiac output. However, if the preload increases excessively, there is an increased risk of worsening heart failure due to impaired myocardial dilatation, as in the first delivery. Therefore, appropriate intraoperative management of the preload is necessary. In the present case, epidural anesthesia was performed, instead of spinal subarachnoid anesthesia to avoid a sudden decrease in preload. While some anesthesia methods for HCM-complicated pregnancies suggest that general anesthesia is safe, others report that regional anesthesia can safely manage the condition [12]. In this case, there was no evidence that general anesthesia was necessary during the third pregnancy, and further studies on anesthesia for HCM-associated pregnancies are needed. Septal myectomy reduces the risk of cardiovascular events by eliminating the factors of left ventricular outflow tract stenosis and mitral regurgitation in the ZAHARA score, as well as those of the left ventricular outflow tract stenosis in the CARPREG II score. Therefore, the ZAHARA and CARPREG II scores are appropriate for evaluation after septal myectomy in pregnancies complicated by HCM.

In the present study, the same patient with HCM underwent different pregnancy outcomes, which were retrospectively evaluated using the three scoring methods: the ZAHARA score, the CARPREG II score, and the modified WHO classification. These scores have been added to the Guidelines for the Indication, Management of Pregnancy, and Childbirth in Patients with Heart Disease [9,13] since its 2018 revision in Japan. These scoring systems may be useful for risk assessment before and during pregnancy, as well as for the consideration of delivery methods.

## Conclusions

Risk assessment using the ZAHARA risk score, CARPREG II score, and modified WHO classification was considered useful in determining the conditions for pregnancy permission and delivery policy for pregnancies complicated with HCM, and in predicting the risk of perinatal cardiovascular events. Although there are limitations to this evaluation since it is based on only one case, these risk scores were able to accurately assess the risk at different times for the same patient. We propose that these indicators be measured before and after pregnancy, periodically during the pregnancy, and as appropriate when symptoms appear. In the future, it will be necessary to conduct prospective evaluations and accumulate data on pregnant women with similar risks. Ultimately, the goal will be to improve the prognosis of mothers and children through interventions based on these evaluations.

## References

[1] Semsarian C, Ingles J, Maron MS, Maron BJ (2015). New perspectives on the prevalence of hypertrophic cardiomyopathy. J Am Coll Cardiol.

[2] Goland S, van Hagen IM, Elbaz-Greener G (2017). Pregnancy in women with hypertrophic cardiomyopathy: data from the European Society of Cardiology initiated Registry of Pregnancy and Cardiac disease (ROPAC). Eur Heart J.

[3] Tanaka H, Kamiya C, Katsuragi S (2014). Cardiovascular events in pregnancy with hypertrophic cardiomyopathy. Circ J.

[4] Choi WY, Park KT, Kim HM (2024). Pregnancy related complications in women with hypertrophic cardiomyopathy: a nationwide population-based cohort study. BMC Cardiovasc Disord.

[5] Sanghavi M, Rutherford JD (2014). Cardiovascular physiology of pregnancy. Circulation.

[6] Pieper PG, Walker F (2013). Pregnancy in women with hypertrophic cardiomyopathy. Neth Heart J.

[7] Drenthen W, Boersma E, Balci A (2010). Predictors of pregnancy complications in women with congenital heart disease. Eur Heart J.

[8] Silversides CK, Grewal J, Mason J (2018). Pregnancy outcomes in women with heart disease: the CARPREG II study. J Am Coll Cardiol.

[9] Regitz-Zagrosek V, Roos-Hesselink JW, Bauersachs J (2018). 2018 ESC Guidelines for the management of cardiovascular diseases during pregnancy. Eur Heart J.

[10] Takase B, Ikeda T, Shimizu W (2024). JCS/JHRS 2022 guideline on diagnosis and risk assessment of arrhythmia. Circ J.

[11] Rosseland LA, Hauge TH, Grindheim G, Stubhaug A, Langesæter E (2013). Changes in blood pressure and cardiac output during cesarean delivery: the effects of oxytocin and carbetocin compared with placebo. Anesthesiology.

[12] Ashikhmina E, Farber MK, Mizuguchi KA (2015). Parturients with hypertrophic cardiomyopathy: case series and review of pregnancy outcomes and anesthetic management of labor and delivery. Int J Obstet Anesth.

[13] (2024). JCS 2018 Guideline on Indication and Management of Pregnancy and Delivery in Women with Heart Disease (In Japanese). JCS.

